# Adverse childhood experiences and handgrip strength among middle-aged and older adults: a cross-sectional study in China

**DOI:** 10.1186/s12877-022-02796-z

**Published:** 2022-02-12

**Authors:** Li Lin, Weidi Sun, Ciyong Lu, Weiqing Chen, Vivian Yawei Guo

**Affiliations:** Department of Epidemiology, School of Public Health, Sun Yat-Sen University, 74 Zhongshan Second Road, Guangzhou, 510080 Guangdong China

**Keywords:** Adverse childhood experiences, Handgrip strength, Middle-aged and older adults, Chinese

## Abstract

**Background:**

Evidence on the association between adverse childhood experiences (ACEs) and handgrip strength (HGS) in later life was limited and inconclusive. We aimed to explore the impact of ACEs on HGS among middle-aged and older Chinese adults.

**Methods:**

We conducted a cross-sectional study with data extracted from the China Health and Retirement Longitudinal Study (CHARLS), a nationally representative survey with respondents recruited from 450 villages/urban communities of 28 provinces. Participants aged 45 years or older were drawn from the CHARLS 2014 life history survey and the 2015 health survey. Twelve ACE indicators before the age of 17 years were collected. HGS was measured with a dynamometer and the maximum value of HGS obtained from both hands was used in the analyses. Low muscle strength (LMS) was defined according to the recommendation of European Working Group on Sarcopenia in Older People (EWGSOP). Multivariate linear and logistic regression models were constructed to evaluate the association of ACEs with continuous HGS and LMS, with adjustment for age, sex, marital status, ethnicity, area of residence, smoking and drinking status, body mass index, hypertension, dyslipidaemia, diabetes mellitus, cardiovascular disease, arthritis, hip fracture, and memory-related disease.

**Results:**

Of the 7209 eligible participants, 2258 (31.3%) had experienced three or more ACEs. Compared to individuals without ACEs, exposure to ≥ 3 ACEs was negatively associated with continuous HGS in kilogram (*β* = -0.93, 95% CI: -1.37, -0.49) and positively associated with the risk of LMS (OR = 1.34, 95% CI: 1.12, 1.61). Such associations were consistently found both in men and women who had experienced three or more ACEs. Significant dose–response relationship between the number of ACEs and outcomes was also observed in the overall population and different sex groups.

**Conclusion:**

Exposure to ACEs was associated with lower HGS and increased risk of LMS among middle-aged and older Chinese adults, indicating the importance of intervention in individuals with experience of ACEs in order to mitigate its detrimental impact on HGS and promote healthy ageing.

**Supplementary Information:**

The online version contains supplementary material available at 10.1186/s12877-022-02796-z.

## Introduction

As a reliable indicator to reflect muscle strength and muscle mass, handgrip strength (HGS) has been widely used to diagnose sarcopenia, a condition characterized with gradual loss of muscle mass and strength [1]. Participants with low muscle strength (LMS) were at higher risk of developing a range of adverse health outcomes, including cognitive impairment, mobility decline, cardiovascular disease, and even mortality [2–5]. Ageing is one of the major risk factors for reduced HGS, with higher prevalence of LMS in older populations [6]. Data from the Korean Longitudinal Study of Aging (KLoSA) demonstrated that the prevalence of LMS in people aged below 65 years was 13.0%, while this figure went up to 67.5% in those aged ≥ 65 years [7]. In addition, low socio-economic status, physical inactivity, malnutrition, and morbidity have also been demonstrated to be risk factors of LMS in later life [8–11].

Adverse childhood experiences (ACEs) are defined as exposure to a string of potentially stressful events during childhood [12]. It is usually comprised of emotional and physical neglect and abuse, household challenges, and other stressful experiences, such as economic hardship, community violence, unsafe neighbourhood, and bullying [12, 13]. Exposure to ACEs has been demonstrated to be a risk factor of adverse health conditions in later life, such as ischemic heart disease, cancer, and chronic lung disease [12, 14]. There is limited research that has also investigated the link between ACEs and LMS in later life, however, with mixed results [8, 15–17]. For example, a cross-sectional study of 24,179 adults aged between 50–96 years from the Survey of Health Ageing and Retirement in Europe (SHARE) found that disadvantaged socioeconomic circumstances in early life were associated with lower muscle strength in later life, especially in women [8]. Data from the same survey further confirmed that compared to women without any ACE exposure, those exposed to two or more ACEs had significantly increased risk of muscle weakness, while such association was not observed in men [15]. On the contrary, the Health and Retirement Study (HRS) showed that experience of childhood misfortune predicted steeper declines in HGS only in men, but not in women [16]. Furthermore, a cross-sectional study of 3732 Chinese adults aged ≥ 65 years even showed that exposure to famine during childhood was not significantly associated with HGS in later life [17]. These limited and inconsistent findings suggested the need for further studies.

In this study, we aimed to assess the association of ACEs with continuous HGS and LMS among middle-aged and older adults using data from the China Health and Retirement Longitudinal Study (CHARLS). Subgroup analysis was also conducted to evaluate the sex-specific associations.

## Methods

### Study design and population

This cross-sectional study used data from the CHARLS, a nationally representative survey of people aged 45 years or older [18]. The baseline survey was conducted from June 2011 to March 2012. A multistage, probability-proportional-to-size (PPS) sampling strategy was used to recruit eligible participants. At first, 150 county-level units were randomly chosen from 28 provinces across China stratified by region, rural/urban residence, and per capita statistics on gross domestic product (GDP). Three neighbourhood communities or administrative villages were then randomly chosen from each county-level unit in urban or rural areas, respectively. In the next step, random sampling was applied to select households from the neighbourhood communities or administrative villages. Collective dwellings, such as military bases, schools, dormitories, nursing homes, and domestic helpers living in the homes of employers were excluded. Then, within each selected household, one person aged 45 years or above was assigned as the main respondent, and her or his spouse was interviewed as well [18]. Participants were followed-up every two years with a small share of new respondents recruited each time. Up to now, three follow-up surveys were conducted in 2013, 2015, and 2018, respectively. Information on life history were additionally collected in 2014 among all live respondents in the first two surveys (i.e., the 2011 and 2013 surveys). In the current study, we used data from the 2014 and 2015 CHARLS surveys, as the most updated data on HGS were collected in 2015.

**Fig. 1:**
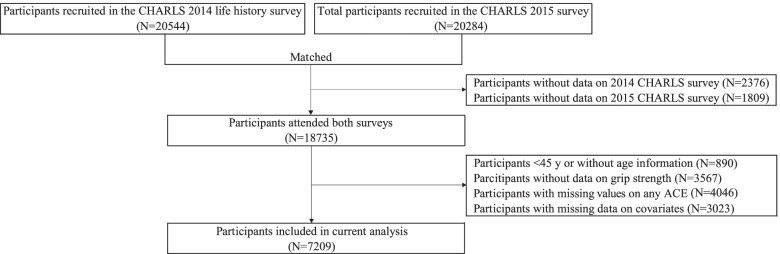
Study flowchart of participant selection

A total of 20,544 and 20,284 individuals were recruited in the CHARLS 2014 and 2015 surveys, respectively (Fig. 1). We identified 18,735 respondents who had attended both surveys. Participants aged < 45 years or without age information (*N* = 890), without data on HGS (*N* = 3567), ACE indicators (*N* = 4046) or covariates (*N* = 3023) were excluded. Finally, 7209 eligible participants aged between 45 and 95 years were included for further analysis on the association between ACEs and HGS.

### Definition of adverse childhood experiences

All ACE indicators were assessed by dichotomous or multiple-choice questions in the CHARLS life history survey (see Supplementary Table 1). Based on previous literatures [13, 19, 20], a total of 12 ACEs before the age of 17 years old were considered in our study, i.e., physical abuse, emotional neglect, household substance abuse, household mental illness, domestic violence, incarcerated household member, parental separation or divorce, unsafe neighbourhood, bullying, parental death, sibling death, and parental disability.

In line with previous studies, participants were further categorized into four groups based on the total number of ACEs they had ever experienced (i.e., 0, 1, 2, and ≥ 3 ACEs) [21, 22].

### Measurement of handgrip strength

HGS was measured by a standardized dynamometer (Yuejian WL-1000, China) in kilograms [23]. Participants were asked to stay in a standing position, hold the dynamometer at a right angle (90°), and squeeze the handle with their maximum effort for a few seconds [23]. Each hand was measured twice and the largest HGS reading obtained from both hands was used in the analysis [24].

We adopted the recommendation of European Working Group on Sarcopenia in Older People (EWGSOP) to define LMS based on sex and body mass index (BMI), which was commonly used in research literatures [15, 25, 26]. In detail, participants were first stratified based on quartiles of BMI in men and women respectively. Then, the lowest 20% of HGS in each stratum were established as the cut-off values for LMS. In our study population, the cut-off points for LMS in men were 28.0, 30.8, 32.0, and 34.0 kg for those with a BMI ≤ 20.8, 20.9–23.1, 23.2–25.7, and > 25.7 kg/m^2^, respectively. The cut-off points for LMS in women were 18.0, 20.5, 21.0, and 21.0 kg for those with a BMI ≤ 21.8, 21.9–24.2, 24.3–26.6, and > 26.6 kg/m^2^, respectively.

### Covariates

Demographic and lifestyle characteristics were collected through face-to-face interviews, including age, sex (men and women), marital status (married/cohabitated and unmarried), ethnicity (Han ethnicity and ethnic minority population), area of residence (rural and urban), smoking (never smoker, ever smoker, and current smoker) and drinking status (never drinker, ever drinker, and current drinker).

BMI was calculated as weight (kg) divided by the square of height (m^2^). Blood pressure was measured three times using an Omron™ HEM-7200 sphygmomanometer [18]. Physician-diagnosed chronic diseases, including hypertension, dyslipidaemia, diabetes mellitus (DM), cardiovascular disease (CVD, including chronic heart disease and stroke), arthritis (including rheumatism), hip fracture, and memory-related disease (including Alzheimer’s disease, Parkinson’s disease, and cerebral atrophy) were self-reported by each participant. Hypertension was confirmed if the participant had physician-diagnosed hypertension, and/or with a mean systolic blood pressure (BP) ≥ 140 mmHg, and/or mean diastolic BP ≥ 90 mmHg, and/or on anti-hypertensive drugs [27]. According to the American Diabetes Association criteria, a participant was defined as having DM if he/she had fasting plasma glucose ≥ 7.0 mmol/L, and/or random plasma glucose ≥ 11.1 mmol/L, and/or glycated hemoglobin (HbA1c) ≥ 6.5%, and/or self-reported DM diagnosed by a physician, and/or on glucose-lowering drugs or insulin treatment [28, 29].

### Statistical analysis

The normality of continuous variable was evaluated by Kolmogorov–Smirnov test combined with Q-Q plot. Comparison of characteristics according to the four categories of ACEs was evaluated by one-way analysis of variance (ANOVA) and Chi-square tests for continuous and categorical variables, respectively. To assess the trend of characteristics across different ACEs groups, ANOVA trend analyses using polynomial contrasts were conducted for continuous data and Mantel–Haenszel test of trend was performed for categorical data.

Multivariate linear and logistic regression models were used to assess the impact of ACEs on continuous HGS (kg) and LMS, respectively. Model 1 was adjusted for age and sex. Model 2 was additionally controlled for marital status, ethnicity, and area of residence. To determine the mediating role of health behaviours and chronic conditions, we further included smoking and drinking status, BMI, hypertension, dyslipidaemia, DM, CVD, arthritis, hip fracture, and memory-related disease in Model 3. Assumptions of linear regression models, including linearity, normality, homoscedasticity and absence of multicollinearity, were checked for all linear regression models. To graphically depict the predicted odds of LMS at different age in the four ACEs categories, we constructed quadratic fitting curves in men and women, respectively, with adjustment for covariates listed in Model 3 [30, 31]. Subgroup analysis was conducted by sex, as sex-specific associations between ACEs and HGS have been reported previously [15]. In consideration of potential survivorship bias, we conducted sensitivity analysis by re-evaluating such association among participants aged < 70 years and those aged ≥ 70 years, respectively.

All analyses were performed using STATA 15.0 (StataCorp. College Station, TX, USA). All tests were two-sided and a *p*-value of less than 0.05 was considered to be statistically significant.

## Results

Of the 7209 participants included in our analysis, 19.8% experienced no ACE and 31.3% reported the experience of three or more ACEs (Table 1). More than half (52.9%) of the participants were women and the mean age was 60.8 (SD: 8.9) years. Compared to participants without ACE exposure, those with experience of three or more ACEs were statistically older, more likely to be men, rural residents, current smokers and drinkers. They also had significantly higher prevalence of CVD, arthritis, and memory-related disease. With increase in the number of ACEs, the mean HGS decreased from 38.6 (SD: 7.7) kg to 36.5 (SD: 8.4) kg in men and from 26.2 (SD: 6.3) kg to 24.5 (SD: 6.6) kg in women. In contrast, the prevalence of LMS increased in both sexes with the number of ACEs increased.

**Table 1:** Characteristics of participants by number of ACEs

**Characteristics**^a^	**Total**	**No. of ACEs**				***p* for difference**	***p* for trend**
		0	1	2	≥ 3		
***n***	7209	1424 (19.8%)	1950 (27.0%)	1577 (21.9%)	2258 (31.3%)		
**Demographic and lifestyle factors**							
Mean age (years)	60.8 (8.9)	60.0 (8.6)	60.4 (8.9)	60.6 (8.8)	61.8 (9.1)	< 0.001	< 0.001
Sex, n (%)						< 0.001	< 0.001
Men	3396 (47.1%)	580 (40.7%)	883 (45.3%)	772 (49.0%)	1161 (51.4%)		
Women	3813 (52.9%)	844 (59.3%)	1067 (54.7%)	805 (51.0%)	1097 (48.6%)		
Marital status, n (%)						0.041	0.015
Married or cohabitated	6353 (88.1%)	1279 (89.8%)	1715 (87.9%)	1399 (88.7%)	1960 (86.8%)		
Unmarried	856 (11.9%)	145 (10.2%)	235 (12.1%)	178 (11.3%)	298 (13.2%)		
Ethnicity, n (%)						0.918	0.499
Han ethnicity	6683 (92.7%)	1316 (92.4%)	1806 (92.6%)	1461 (92.6%)	2100 (93.0%)		
Ethnic minority population	526 (7.3%)	108 (7.6%)	144 (7.4%)	116 (7.4%)	158 (7.0%)		
Area of residence, n (%)						0.004	0.001
Rural	4660 (64.6%)	897 (63.0%)	1224 (62.8%)	1013 (64.2%)	1526 (67.6%)		
Urban	2549 (35.4%)	527 (37.0%)	726 (37.2%)	564 (35.8%)	732 (32.4%)		
Smoking status, n (%)						< 0.001	< 0.001
Never smoker	4110 (57.0%)	906 (63.6%)	1146 (58.8%)	854 (54.2%)	1204 (53.3%)		
Ever smoker	999 (13.9%)	172 (12.1%)	260 (13.3%)	239 (15.2%)	328 (14.5%)		
Current smoker	2100 (29.1%)	346 (24.3%)	544 (27.9%)	484 (30.7%)	726 (32.2%)		
Drinking status, n (%)						< 0.001	< 0.001
Never drinker	3884 (53.9%)	880 (61.8%)	1100 (56.4%)	788 (50.0%)	1116 (49.4%)		
Ever drinker	809 (11.2%)	125 (8.8%)	211 (10.8%)	180 (11.4%)	293 (13.0%)		
Current drinker	2516 (34.9%)	419 (29.4%)	639 (32.8%)	609 (38.6%)	849 (37.6%)		
**Health assessment**							
BMI (kg/m^2^)	23.9 (3.8)	24.2 (3.8)	24.0 (3.7)	24.1 (3.8)	23.6 (3.8)	< 0.001	< 0.001
Systolic BP (mmHg)	128.1 (19.9)	127.7 (19.7)	128.5 (19.4)	129.2 (20.6)	127.4 (20.1)	0.037	0.520
Diastolic BP (mmHg)	75.6 (11.8)	75.5 (11.9)	75.5 (11.4)	76.5 (12.0)	74.9 (11.9)	< 0.001	0.289
**History of chronic diseases, n (%)**							
Hypertension	3109 (43.1%)	598 (42.0%)	832 (42.7%)	698 (44.3%)	981 (43.4%)	0.608	0.303
Dyslipidaemia	1172 (16.3%)	217 (15.2%)	298 (15.3%)	270 (17.1%)	387 (17.1%)	0.203	0.052
DM	1265 (17.5%)	248 (17.4%)	320 (16.4%)	280 (17.8%)	417 (18.5%)	0.372	0.196
CVD	1314 (18.2%)	252 (17.7%)	318 (16.3%)	279 (17.7%)	465 (20.6%)	0.003	0.004
Arthritis	2704 (37.5%)	425 (29.8%)	634 (32.5%)	612 (38.8%)	1033 (45.7%)	< 0.001	< 0.001
Hip fracture	149 (2.1%)	25 (1.8%)	40 (2.1%)	26 (1.6%)	58 (2.6%)	0.183	0.129
Memory-related disease	168 (2.3%)	29 (2.0%)	34 (1.7%)	37 (2.3%)	68 (3.0%)	0.044	0.014
**HGS (kg)**							
Men	37.7 (8.5)	38.6 (7.7)	38.4 (8.9)	38.1 (8.5)	36.5 (8.4)	< 0.001	< 0.001
Women	25.5 (6.5)	26.2 (6.3)	25.8 (6.5)	25.7 (6.5)	24.5 (6.6)	< 0.001	< 0.001
**LMS, n (%)**							
Men	695 (20.5%)	94 (16.2%)	168 (19.0%)	145 (18.8%)	288 (24.8%)	< 0.001	< 0.001
Women	788 (20.7%)	150 (17.8%)	205 (19.2%)	158 (19.6%)	275 (25.1%)	< 0.001	< 0.001

*ACE* adverse childhood experience, *BMI* body mass index, *BP* blood pressure, *CVD* cardiovascular disease, *DM* diabetes mellitus, *HGS* handgrip strength, *LMS* low muscle strength

^a^Continuous data were reported as mean (SD) and categorical data were reported as number (percentage)

The associations between ACEs with continuous HGS were presented in Table 2. In the overall population, compared to participants with no ACE exposure, those experienced three or more ACEs had significantly lower HGS in Model 1 (*β* = -1.26, 95% CI: -1.71, -0.81). We also observed a significantly decreasing trend in HGS as the number of ACEs increased (*p* value for trend < 0.001). Similar associations were found in the adjusted Model 2. After further adjustment for health behaviours and chronic diseases in Model 3, the effect size of the association between ACEs and HGS was attenuated but still significant among adults with exposure to three or more ACEs (*β* = -0.93, 95% CI: -1.37, -0.49). Similar findings were also observed for the ACE ≥ 3 group in both men (Model 3, *β* = -1.04, 95% CI: -1.75, -0.33; *p* value for trend = 0.001) and women (Model 3, *β* = -0.93, 95% CI: -1.46, -0.39; *p* value for trend = 0.001).

**Table 2:** Association between ACEs and continuous HGS (kg) in the overall population and different sex groups in linear regression models

	**Overall**^**d**^ ***N* = 7209**	**Men**^**d**^ ***N* = 3396**	**Women**^**d**^ ***N* = 3813**
**Model 1**^a^	***β* (95% CI)**		
** ACEs**			
0	ref	ref	ref
1	-0.16 (-0.62, 0.30)	-0.19 (-0.97, 0.58)	-0.22 (-0.76, 0.32)
2	-0.34 (-0.82, 0.14)	-0.39 (-1.18, 0.40)	-0.36 (-0.94, 0.21)
≥ 3	-1.26 (-1.71, -0.81)	-1.39 (-2.12, -0.65)	-1.17 (-1.71, -0.64)
* p* for trend	< 0.001	< 0.001	< 0.001
**Model 2**^b^			
** ACEs**			
0	ref	ref	ref
1	-0.16 (-0.61, 0.30)	-0.20 (-0.96, 0.56)	-0.21 (-0.75, 0.32)
2	-0.32 (-0.80, 0.16)	-0.39 (-1.17, 0.39)	-0.35 (-0.93, 0.22)
≥ 3	-1.18 (-1.62, -0.73)	-1.33 (-2.06, -0.61)	-1.11 (-1.65, -0.57)
* p* for trend	< 0.001	< 0.001	< 0.001
**Model 3**^c^			
** ACEs**			
0	ref	ref	ref
1	-0.12 (-0.57, 0.32)	-0.22 (-0.96, 0.51)	-0.18 (-0.71, 0.35)
2	-0.28 (-0.75, 0.19)	-0.40 (-1.16, 0.36)	-0.29 (-0.87, 0.28)
≥ 3	-0.93 (-1.37, -0.49)	-1.04 (-1.75, -0.33)	-0.93 (-1.46, -0.39)
* p* for trend	< 0.001	0.001	0.001

*ACE* adverse childhood experience, *CI* confidence interval, *HGS* handgrip strength

^a^Model 1: Adjusted for age and sex in the overall population, and adjusted for age in different sex groups

^b^Modle 2: Additionally adjusted for marital status, ethnicity, and area of residence

^c^Model 3: Additionally adjusted for smoking and drinking status, BMI, hypertension, dyslipidaemia, DM, CVD, arthritis, hip fracture, and memory-related disease

^d^In the overall population, the average HGS were 31.2, 31.5, 31.7, and 30.7 kg in the groups with 0, 1, 2, and ≥ 3 ACEs. In men, the average HGS were 38.6, 38.4, 38.1, and 36.5 kg in the groups with 0, 1, 2, and ≥ 3 ACEs. In women, the average HGS were 26.2, 25.8, 25.7, and 24.5 kg in the groups with 0, 1, 2, and ≥ 3 ACEs

We further assessed the associations between ACEs and the prevalence of LMS in the overall population and by sex (Table 3). Compared to participants without any exposure to ACE, those with three or more ACEs had significantly increased risk of LMS in all models (OR = 1.44, 95% CI: 1.21, 1.72 in Model 1, OR = 1.41, 95% CI: 1.18, 1.68 in Model 2, and OR = 1.34, 95% CI: 1.12, 1.61 in Model 3). However, exposure to one or two ACEs was not significantly associated with the prevalence of LMS in any of the models. Additionally, compared to the reference group, both men and women with three or more ACE exposures had increased likelihood of having LMS even in the fully adjusted model (Model 3, OR = 1.48, 95% CI: 1.11, 1.96 in men, and OR = 1.30, 95% CI: 1.02, 1.64 in women).

**Table 3:** Association between ACEs and LMS in the overall population and different sex groups in logistic regression models

	**Overall**^**d**^ ***N* = 7209**	**Men**^**d**^ ***N* = 3396**	**Women**^**d**^ ***N* = 3813**
**Model 1**^a^	**OR (95% CI)**		
** ACEs**			
0	ref	ref	ref
1	1.11 (0.92, 1.34)	1.24 (0.93, 1.67)	1.04 (0.81, 1.32)
2	1.11 (0.91, 1.35)	1.18 (0.87, 1.60)	1.07 (0.83, 1.39)
≥ 3	1.44 (1.21, 1.72)	1.56 (1.18, 2.05)	1.37 (1.09, 1.73)
* p* for trend	< 0.001	0.002	0.004
**Model 2**^b^			
** ACEs**			
0	ref	ref	ref
1	1.10 (0.91, 1.33)	1.24 (0.92, 1.67)	1.03 (0.81, 1.32)
2	1.10 (0.90, 1.34)	1.18 (0.87, 1.61)	1.07 (0.83, 1.38)
≥ 3	1.41 (1.18, 1.68)	1.54 (1.17, 2.03)	1.35 (1.07, 1.70)
* p* for trend	< 0.001	0.002	0.007
**Model 3**^c^			
** ACEs**			
0	ref	ref	ref
1	1.10 (0.91, 1.33)	1.27 (0.94, 1.72)	1.03 (0.80, 1.31)
2	1.08 (0.89, 1.32)	1.17 (0.86, 1.60)	1.05 (0.81, 1.36)
≥ 3	1.34 (1.12, 1.61)	1.48 (1.11, 1.96)	1.30 (1.02, 1.64)
* p* for trend	0.001	0.011	0.023

*ACE* adverse childhood experience, *CI* confidence interval, *LMS* low muscle strength, *OR* odds ratio

^a^Model 1: Adjusted for age and sex in the overall population, and adjusted for age in different sex groups

^b^Model 2: Additionally adjusted for marital status, ethnicity, and area of residence

^c^Model 3: Additionally adjusted for smoking and drinking status, BMI, hypertension, dyslipidaemia, DM, CVD, arthritis, hip fracture, and memory-related disease

^d^In the overall population, the prevalence of LMS was 17.1%, 19.1%, 19.2%, and 24.9% in the groups with 0, 1, 2, and ≥ 3 ACEs. In men, the prevalence of LMS was 16.2%, 19.0%, 18.8%, and 24.8% in the groups with 0, 1, 2, and ≥ 3 ACEs. In women, the prevalence of LMS was 17.8%, 19.2%, 19.6%, and 25.1% in the groups with 0, 1, 2, and ≥ 3 ACEs

**Fig. 2:**
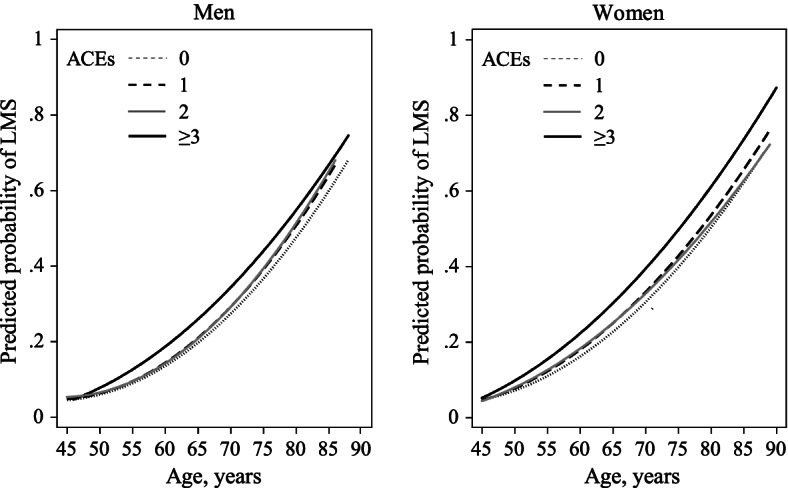
Predicted probability of LMS by number of ACEs and sex. ACE, adverse childhood experience; LMS, low muscle strength

Figure 2 presented the sex-specific predicted probability of LMS at different age in four levels of ACEs. The predicted probability of LMS increased with age in both sexes. Compared to those reported no experience of ACEs, the odds of LMS increased in those with ACE exposures and was the highest in the group with exposure to three or more ACEs.

In sensitivity analysis, the impact of ACEs on HGS remained evident among those aged < 70 years, whereas it was attenuated to statistically insignificant among participants aged ≥ 70 years (Supplementary Table 2 and 3).

## Discussion

In this cross-sectional study, we found that exposure to three or more ACEs was associated with lower HGS and increased risk of LMS among middle-aged and older adults. The associations were statistically significant in both men and women. In addition, there was a significant dose–response relationship between the number of ACEs and risk of LMS. We further observed that the risk of LMS increased with age, in both sex groups.

The associations of ACEs with HGS have not been well documented before [8, 15–17]. One comparable cross-sectional analysis of the SHARE study was conducted among participants aged between 50–96 years from 14 European countries [15]. It suggested that exposure to two or more ACEs was positively associated with the risk of muscle weakness in women, but not in men [15]. Similar findings were also reported in the association between disadvantaged early-life socioeconomic circumstances and later-life muscle strength from the same study [8]. In contrast, a study conducted in the United States found that childhood misfortune was linked to lower HGS in both sexes in the cross-sectional settings, while the longitudinal analysis revealed that childhood misfortune was associated with steeper declines in HGS only in men, but not in women [16]. A Chinese study even showed that famine during childhood was not significantly associated with lower HGS among older people ≥ 65 years [17].

The inconsistent findings across different studies could be explained by discrepancies in ACE definitions. Our study covered a relatively wider range of adverse events during childhood and further categorized the 12 ACEs into one of four cumulative-score groups, while other studies captured different domains of childhood adversities, such as childhood disease and impairment [16], or only evaluated the impact of a single ACE indicator on HGS [8, 17]. Nevertheless, measuring a single ACE component might overestimate its impact on HGS [16], as previous evidence suggested that ACE indicators were interrelated and usually happened in clusters [32, 33]. Therefore, evaluating associations between the cumulative number of ACEs experienced and HGS might be more clinically significant. Furthermore, HGS measurement was also diverse across different studies. Our study used the maximum value of HGS obtained from two hands in the analyses based on a well-established protocol for measuring HGS in large epidemiological studies [24], while some studies used the mean value of HGS collected from both hands [16, 17]. Another explanation for the discrepancies across studies might be the different socio-cultural backgrounds, as individual’s perception and reaction of ACEs could be distinct in different cultures [34]. Future studies could further compare the impact of ACEs on health outcomes across different cultures. Therefore, it was reasonable for the inconsistent findings in different studies.

The exact mechanisms underlying the association between ACE exposure and low HGS are so far not fully understood. Individuals with exposure to ACEs had chronic stress and elevated allostatic load [35–37], which could further lead to increased inflammation levels and dysfunction in the immune system [35, 38], and subsequently cause damages in skeletal muscle and lower HGS [39]. Previous evidence has also suggested a positive association between ACE exposures and telomere length shortening [40], which could lead to cellular dysfunction in tissues and organs, and ultimately result in declines in HGS [41]. Furthermore, stress caused by ACEs might impair the development of brain and nervous system, which could further lead to problems in coping, planning, learning, and self-management [36, 42, 43]. This provided some explanation for the associations between ACEs and several behavioural problems in adulthood, such as smoking, drinking, physical inactivity, and sleep disorders [12, 44–46]. These behavioural problems could consequently deteriorate musculoskeletal health and cause LMS [47–49]. In our study, we also observed a positive trend between the number of ACEs experienced and the prevalence of smoking and drinking. However, after further adjustment for the lifestyle factors and chronic diseases, the significant association between ACEs and HGS was still present, suggesting that ACEs was an independent risk factor for declines in HGS.

When explored such association by age, the deleterious impact of ACEs on HGS among participants aged ≥ 70 years was attenuated to statistical insignificance. This might be explained by the potential survivorship bias, as the vulnerable population exposed to greater ACEs might have died earlier than those without ACE exposure. Therefore, individuals aged ≥ 70 years in our study could be derived from the most resilient survivors who were less likely to be affected by ACEs. Nevertheless, future longitudinal studies could reduce such bias by addressing censored data appropriately.

To our best knowledge, this was among the first studies to evaluate the association between the cumulative adversities during childhood and HGS in later life among middle-aged and older Chinese. We not only assessed the impact of ACEs on continuous HGS, but also defined LMS based on age- and BMI-specific cut-off points according to well-accepted recommendations [25]. We have also included different domains of ACEs, which all have been proven to be significant risk factors for several health outcomes [12, 20]. However, our study has some limitations. First, information on ACEs was retrospectively collected through self-report, which was subjective to recall bias. Previous literatures have indicated that prospective and retrospective measures of ACEs could identify different groups of individuals [50, 51]. In addition, retrospective measures might underestimate the impact of ACEs on objectively evaluated health outcomes, like HGS [51]. Nevertheless, retrospective reports of major and easily defined ACEs are considered to have acceptable psychometric properties, and might provide some unique and complementary information compared to prospective measurement of ACEs [51, 52]. Therefore, retrospective studies on ACEs still have a worthwhile place in epidemiological research and clinical practice [50, 52]. Second, although we have adjusted for several confounders in the multivariate analyses, some reported risk factors of LMS, such as physical activity and diet [53, 54], were not included due to data unavailability. Third, resilience was found to be an effect modifier in the association between ACEs and multiple health outcomes in adulthood [55, 56]. Nonetheless, we are unable to explore the modifying role of resilience, since no relevant information was collected in CHARLS. Future studies should confirm the possible role of resilience in reducing the burden of LMS among individuals with exposure to ACEs.

## Conclusion

This study demonstrated the significant associations of exposure to ACEs with lower HGS and increased likelihood of LMS among middle-aged and older Chinese adults, without sex differences. Significant dose–response relationship was observed between the number of ACE exposures and the outcomes. Our findings add fuel to the ongoing research about the lifelong adverse effects of ACEs on health outcomes. It also emphasized the importance of intervening individuals with ACEs, in order to mitigate its detrimental impact on HGS and promote healthy ageing. Nonetheless, future longitudinal studies and biomolecular research are required to confirm such associations and underlying mechanisms.

## Supplementary Information

**Additional file 1: Table 1.** Definition of adverse childhood experiences. **Table 2.** Association between ACEs and continuous HGS (kg) by age in the overall population and different sex groups in linear regression models.** Table 3. **Association between ACEs and LMS by age in the overall population and different sex groups in logistic regression models.

## Data Availability

The data that support the findings of this study are available on the website of the China Health and Retirement Longitudinal Study (CHARLS) at http://charls.pku.edu.cn/index/en.html. To access and use this survey data for research purpose, an approval should be obtained from the CHARLS team at Peking University.
