# Correction: Adverse childhood experiences and handgrip strength among middle-aged and older adults: a cross-sectional study in China

**DOI:** 10.1186/s12877-022-03159-4

**Published:** 2022-06-28

**Authors:** Li Lin, Weidi Sun, Ciyong Lu, Weiqing Chen, Vivian Yawei Guo

**Affiliations:** Department of Epidemiology, School of Public Health, Sun Yat-Sen University, 74 Zhongshan Second Road, Guangzhou, 510080 Guangdong China

**Correction: BMC Geriatr 22, 118 (2022)**

**https://doi.org/10.1186/s12877-022-02796-z**

After publication of this article [1], the authors reported that in Fig. 1, the number of participants without data in the CHARLS 2014 survey should have been 1549 (instead of 2376); the figure should have appeared as shown below.

**Fig. 1:**
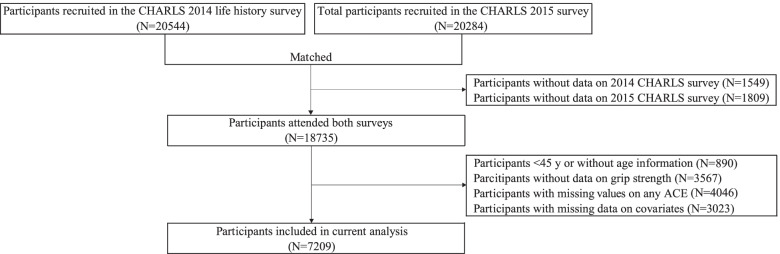
Study flowchart of participant selection

The original article [1] has been updated.
