# Supplementary material for: Adverse childhood experiences and handgrip strength among middle-aged and older adults: a cross-sectional study in China
Source: BMC Geriatr. 2022 Feb 12;22:118. doi: 10.1186/s12877-022-02796-z (PMC8840034; doi:10.1186/s12877-022-02796-z)
Supplement: Supplementary file 1 — Additional file 1: Table 1. Definition of adverse childhood experiences. Table 2. Association between ACEs and continuous HGS (kg) by age in the overall population and different sex groups in linear regression models. Table 3. Association between ACEs and LMS by age in the overall population and different sex groups in logistic regression models. [file 12877_2022_2796_MOESM1_ESM.docx]

**Supplementary Table 1. Definition of adverse childhood experiences.**

| **Domains of ACEs** | **Questionnaire Items** | **Answers defined as exposure to the domain** |
| --- | --- | --- |
| Physical abuse | When you were growing up, did your female/male guardian ever hit you? | often/sometimes |
| Emotional Neglect | How much love and affection did your female guardian give you while you were growing up? | rarely/never |
|  | How much effort did your female guardian put into watching over you? | a little/not at all |
| Household substance abuse | During the years you were growing up, which one of the followings did your female guardian ever have? | alcoholism and/or drug |
| Household mental illness | Did your female/male guardian have abnormality of mind when you were young? | yes |
|  | During the years you were growing up, had your female/male guardian showed continued signs of sadness or depression that lasted two weeks or more? | yes |
|  | Was this problem of your female/male guardian, sadness or depression during all, most, some, or only a little of your childhood? | all/most |
| Domestic violence | Have your father/mother ever beat up your mother/father? | often/sometimes |
| Incarcerated household member | During the years you were growing up, which one of the followings did your female/male guardian ever have? | arrested/sent to prison |
| Parental separation or divorce | Were your biological parents divorced (including long separation due to emotional problems) before you were 17 years? | yes |
|  |  |  |
| Unsafe neighborhood | Was it safe being out alone at night in the neighborhood where you lived as a child? | not very safe/not safe at all |
| Bullying | When you were a child, how often were you picked on or bullied by kids in your neighborhood? | often/sometimes |
|  | When you were a child, how often were you picked on or bullied by kids in your school? | often/sometimes |
|  |  |  |
| Parental death^a^ | Either of the parents was dead before participant was 17 years. | yes |
| Sibling death^b^ | Any of the siblings was dead before participant was 17 years. | yes |
| Parental disability | Did your female guardian have a long time be sick on bed when you were young? | yes |
|  | Did your female guardian have a serious deformity when you were young? | yes |

Abbreviation: ACEs: Adverse Childhood Experiences.

^a^Calculated based on dates of birth and their parental death.

^b^Calculated based on dates of birth and their sibling’s death.

**Supplementary Table 2. Association between ACEs and continuous HGS (kg) by age in the overall population and different sex groups in linear regression models.**

|  | | **<70 years (N = 5946)** | | |  | **≥70 years (N = 1263)** | | |
| --- | --- | --- | --- | --- | --- | --- | --- | --- |
|  |  | **Overall** | **Men** | **Women** |  | **Overall** | **Men** | **Women** |
|  |  | ***β* (95%CI)** | | |  | ***β* (95%CI)** | | |
| **Model 1**^a^ | |  | | |  |  | | |
| **ACEs** | |  |  |  |  |  |  |  |
|  | 0 | ref | ref | ref |  | ref | ref | ref |
|  | 1 | -0.20 (-0.72, 0.33) | -0.05 (-0.97, 0.86) | -0.31 (-0.92, 0.29) |  | 0.23 (-0.94, 1.39) | 0.11 (-1.67, 1.90) | 0.34 (-1.14, 1.82) |
|  | 2 | -0.47 (-1.02, 0.08) | -0.41 (-1.35, 0.53) | -0.52 (-1.17, 0.12) |  | -0.05 (-1.28, 1.17) | -0.15 (-1.98, 1.67) | 0.06 (-1.55, 1.67) |
|  | ≥3 | -1.65 (-2.17, -1.14) | -1.82 (-2.70, -0.95) | -1.49 (-2.09, -0.88) |  | -1.00 (-2.11, 0.11) | -1.08 (-2.74, 0.58) | -0.92 (-2.37, 0.52) |
|  | *p* for trend | <0.001 | <0.001 | <0.001 |  | 0.019 | 0.093 | 0.095 |
| **Model 2**^b^ | |  | | |  |  | | |
| **ACEs** | |  |  |  |  |  |  |  |
|  | 0 | ref | ref | ref |  | ref | ref | ref |
|  | 1 | -0.19 (-0.71, 0.33) | -0.11 (-1.01, 0.79) | -0.29 (-0.89, 0.31) |  | 0.36 (-0.80, 1.51) | 0.32 (-1.44, 2.08) | 0.38 (-1.10, 1.86) |
|  | 2 | -0.46 (-1.00, 0.09) | -0.49 (-1.41, 0.44) | -0.49 (-1.13, 0.16) |  | 0.12 (-1.10, 1.34) | 0.03 (-1.77, 1.84) | 0.12 (-1.50, 1.73) |
|  | ≥3 | -1.53 (-2.04, -1.03) | -1.79 (-2.65, -0.93) | -1.36 (-1.96, -0.76) |  | -0.85 (-1.95, 0.25) | -0.92 (-2.56, 0.72) | -0.87 (-2.32, 0.59) |
|  | *p* for trend | <0.001 | <0.001 | <0.001 |  | 0.034 | 0.116 | 0.113 |
| **Model 3**^c^ | |  | | |  |  | | |
| **ACEs** | |  |  |  |  |  |  |  |
|  | 0 | ref | ref | ref |  | ref | ref | ref |
|  | 1 | -0.12 (-0.62, 0.39) | -0.10 (-0.97, 0.76) | -0.20 (-0.79, 0.40) |  | 0.22 (-0.90, 1.35) | -0.03 (-1.69, 1.63) | 0.33 (-1.16, 1.83) |
|  | 2 | -0.37 (-0.90, 0.16) | -0.48 (-1.37, 0.41) | -0.35 (-0.98, 0.29) |  | 0.18 (-1.01, 1.36) | 0.14 (-1.57, 1.84) | -0.04 (-1.67, 1.59) |
|  | ≥3 | -1.18 (-1.68, -0.68) | -1.38 (-2.22, -0.55) | -1.03 (-1.64, -0.43) |  | -0.52 (-1.60, 0.56) | -0.64 (-2.20, 0.91) | -0.95 (-2.46, 0.56) |
|  | *p* for trend | <0.001 | <0.001 | 0.001 |  | 0.198 | 0.339 | 0.104 |

Abbreviation: ACE, adverse childhood experience; CI, confidence interval; HGS, handgrip strength.

^a^Model 1: Adjusted for sex in the overall population; crude model in different sex groups.

^b^Model 2: Additionally adjusted for marital status, ethnicity, and area of residence.

^c^Model 3: Additionally adjusted for smoking and drinking status, BMI, hypertension, dyslipidemia, DM, CVD, arthritis, hip fracture, and memory-related disease.

**Supplementary Table 3. Association between ACEs and LMS by age in the overall population and different sex groups in logistic regression models.**

|  | | **<70 years (N = 5946)** | | |  | **≥70 years (N = 1263)** | | |
| --- | --- | --- | --- | --- | --- | --- | --- | --- |
|  |  | **Overall** | **Men** | **Women** |  | **Overall** | **Men** | **Women** |
|  |  | **OR (95% CI)** | | |  | **OR (95% CI)** | | |
| **Model 1**^a^ | |  | | |  |  | | |
| **ACEs** | |  |  |  |  |  |  |  |
|  | 0 | ref | ref | ref |  | ref | ref | ref |
|  | 1 | 1.14 (0.92, 1.42) | 1.22 (0.85, 1.74) | 1.09 (0.82, 1.45) |  | 0.98 (0.69, 1.39) | 1.17 (0.71, 1.93) | 0.83 (0.51, 1.35) |
|  | 2 | 1.23 (0.98, 1.55) | 1.17 (0.81, 1.69) | 1.28 (0.96, 1.72) |  | 0.88 (0.61, 1.27) | 1.19 (0.71, 1.98) | 0.64 (0.38, 1.10) |
|  | ≥3 | 1.71 (1.39, 2.10) | 1.69 (1.22, 2.36) | 1.72 (1.32, 2.24) |  | 1.13 (0.81, 1.57) | 1.52 (0.95, 2.41) | 0.83 (0.51, 1.34) |
|  | *p* for trend | <0.001 | 0.001 | <0.001 |  | 0.402 | 0.06 | 0.434 |
| **Model 2**^b^ | |  | | |  |  | | |
| **ACEs** | |  |  |  |  |  |  |  |
|  | 0 | ref | ref | ref |  | ref | ref | ref |
|  | 1 | 1.14 (0.91, 1.42) | 1.23 (0.86, 1.77) | 1.09 (0.82, 1.45) |  | 0.96 (0.67, 1.36) | 1.15 (0.69, 1.90) | 0.81 (0.50, 1.33) |
|  | 2 | 1.23 (0.98, 1.55) | 1.20 (0.83, 1.73) | 1.28 (0.95, 1.71) |  | 0.85 (0.59, 1.23) | 1.18 (0.70, 1.97) | 0.62 (0.36, 1.07) |
|  | ≥3 | 1.66 (1.35, 2.04) | 1.70 (1.22, 2.37) | 1.67 (1.28, 2.19) |  | 1.10 (0.79, 1.54) | 1.48 (0.93, 2.36) | 0.80 (0.50, 1.30) |
|  | *p* for trend | <0.001 | 0.001 | <0.001 |  | 0.482 | 0.073 | 0.377 |
| **Model 3**^c^ | |  | | |  |  | | |
| **ACEs** | |  |  |  |  |  |  |  |
|  | 0 | ref | ref | ref |  | ref | ref | ref |
|  | 1 | 1.14 (0.91, 1.42) | 1.29 (0.89, 1.86) | 1.06 (0.80, 1.42) |  | 0.94 (0.66, 1.35) | 1.17 (0.69, 1.97) | 0.79 (0.48, 1.30) |
|  | 2 | 1.18 (0.94, 1.49) | 1.20 (0.82, 1.75) | 1.19 (0.88, 1.60) |  | 0.84 (0.57, 1.22) | 1.11 (0.65, 1.90) | 0.67 (0.39, 1.16) |
|  | ≥3 | 1.55 (1.25, 1.91) | 1.61 (1.15, 2.27) | 1.54 (1.17, 2.02) |  | 1.06 (0.75, 1.49) | 1.46 (0.89, 2.39) | 0.85 (0.51, 1.41) |
|  | *p* for trend | <0.001 | 0.008 | 0.001 |  | 0.672 | 0.119 | 0.589 |

Abbreviation: ACE, adverse childhood experience; CI, confidence interval; LMS, low muscle strength; OR, odds ratio.

^a^Model 1: Adjusted for sex in the overall population; crude model in different sex groups.

^b^Model 2: Additionally adjusted for marital status, ethnicity, and area of residence.

^c^Model 3: Additionally adjusted for smoking and drinking status, BMI, hypertension, dyslipidemia, DM, CVD, arthritis, hip fracture, and memory-related disease.
